# Supplementary material for: Chromothripsis during telomere crisis is independent of NHEJ, and consistent with a replicative origin
Source: Genome Res. 2019 May;29(5):737–49. doi: 10.1101/gr.240705.118 (PMC6499312; doi:10.1101/gr.240705.118)
Supplement: Supplemental Material [file supp_gr.240705.118_Supplemental_file_1.zip › contigs/annotated_contigs/DB50/contig.2.DB50_length_289_mean_cov_3.63321799308.docx]

**DB50_length_289_mean_cov_3.63321799308**

```
GTGAGCAAGGTGCAGGGCACAGGGACAACATGGGATGCAGCCCGGGCTCCATTTCCCACTGGCCCTCCATGAAGGGAGTGGCACCCACT
 >chr17:74145358-74145587 + E=7e-127
TGAAGAGGGAAATAGACTCAAGCAGAGACCAGGCGCATGTGCAGCTGTTCTGACGGGAGAGAGCTGGCCACAGGAGCAATGAGGCGCCA

AGACGGCCAGTGGTATCTCAGCCCCAGGATAGAAAGCTGAACTTACAG|CTG|GCACAAACTGTGAGCCCACCATAATCTCTAAATAGC
                                                 >chr17:74146358-74146421 + E=1e-26
TGGCTCCACAAGTCACCCTAGGGA
```
